# Leukocyte immunoglobulin-like receptor B4 deficiency exacerbates acute lung injury via NF-κB signaling in bone marrow-derived macrophages

**DOI:** 10.1042/BSR20181888

**Published:** 2019-06-14

**Authors:** Tao Qiu, Jiangqiao Zhou, Tianyu Wang, Zhongbao Chen, Xiaoxiong Ma, Long Zhang, Jilin Zou

**Affiliations:** Department of Organ Transplantation, Renmin Hospital of Wuhan University, Wuhan University, Wuhan 430060, China

**Keywords:** acute lung injury, LILRB4, lung, macrophage

## Abstract

Acute lung injury (ALI) is an acute inflammatory disease. Leukocyte immunoglobulin-like receptor B4 (LILRB4) is an immunoreceptor tyrosine-based inhibitory motif (ITIM)-bearing inhibitory receptor that is implicated in various pathological processes. However, the function of LILRB4 in ALI remains largely unknown. The aim of the present study was to explore the role of LILRB4 in ALI. LILRB4 knockout mice (LILRB4 KO) were used to construct a model of ALI. Bone marrow cell transplantation was used to identify the cell source of the LILRB4 deficiency-aggravated inflammatory response in ALI. The effect on ALI was analyzed by pathological and molecular analyses. Our results indicated that LILRB4 KO exacerbated ALI triggered by LPS. Additionally, LILRB4 deficiency can enhance lung inflammation. According to the results of our bone marrow transplant model, LILRB4 regulates the occurrence and development of ALI by bone marrow-derived macrophages (BMDMs) rather than by stromal cells in the lung. The observed inflammation was mainly due to BMDM-induced NF-κB signaling. In conclusion, our study demonstrates that LILRB4 deficiency plays a detrimental role in ALI-associated BMDM activation by prompting the NF-κB signal pathway.

## Introduction

Acute lung injury (ALI) is a serious respiratory illness with a high mortality. It results in acute diffuse inflammatory lung tissue injury that is caused by various pathogenic factors [1]; however, the pathogenesis of ALI has not been fully characterized [2]. At present, one of the main causes of ALI pathogenesis is thought to be an imbalance between natural immunity and inflammatory responses [3]. During the ALI inflammatory process, cytokines such as TNF-α, IL-1, IL-10, ICAM-1, MCP-1, and CXCL2 are expressed and secreted [4–6]. In addition, the inflammatory cytokines and chemokines can recruit neutrophils and other inflammatory cells to further release inflammatory factors and cytokines that promote inflammation in the lungs. Moreover, harmful molecules, such as proteolytic enzymes and reactive oxygen species, damage pulmonary capillary endothelial cells and alveolar epithelial cells, leading to the development of ALI. There are no specific therapeutic measures and specific drugs for this disease [7]. Therefore, it is important to discover new targets to treat inflammation in ALI.

Leukocyte Ig-like receptor (LILR) family proteins are expressed by bone marrow mononuclear cells and lymphocytes, which extend their influence across antigen-presenting cell subsets [8]. They have been identified as important players in response to infection and inflammatory diseases [9]. LILR subfamily B member 4 (LILRB4) is a member of the LILR family [10]. LILRB4 is mainly expressed on the surface of antigen-presenting cells, such as dendritic cells and macrophages, and is also expressed in vascular endothelial cells. The extracellular region of LILRB4 contains two c2-type structural domains that can combine with ligands, and the cytoplasmic region contains four immunoreceptor tyrosine-based inhibitory motifs (ITIMs) [11]. LILRB4 is an important natural immune regulatory receptor that is involved in the occurrence and development of various diseases through the regulation of natural immunity and inflammatory reactions [12–14]. However, it is not clear whether LILRB4 plays an important regulatory role in the occurrence and development of ALI.

In the present study, using LILRB4 knockout mice (LILRB4 KO), we found that the LILRB4 expression was up-regulated in our model of ALI, and furthermore, LILRB4 deficiency enhanced the inflammatory response in ALI. In further studies, we found that the exaggerated inflammatory response was mainly due to macrophages via activation of the NF-κB signaling pathway.

## Materials and methods

### Reagents

The antibody against LILRB4 was purchased from GeneTex (GTX33296). The antibodies against p-IkBα (9246), IkBα (4814), p65 (4764), p-p65 (3033), p-Ikkβ (2078), Ikkβ (8943), and GAPDH (2118) were purchased from Cell Signaling Technology. The goat anti-mouse (115-035-003) and goat anti-rabbit (111-035-003) secondary antibodies were purchased from Jackson Laboratory. The BCA protein assay kit was purchased from Pierce. Fetal calf serum (FCS) was obtained from HyClone. The cell culture reagents and all other reagents were obtained from Sigma.

### Animal preparation and experimental design

All the animal experimental protocols were approved by the Animal Care and Use Committee of Renmin Hospital of Wuhan University. The study was approved by the Ethics Committee of Renmin Hospital of Wuhan University. Animal maintenance was in accordance with the standard guidelines of the Animal Experiment Center of Wuhan University. We conducted experiments following the National Institutes of Health (NIH) Guide for the Care and Use of Laboratory Animals. Mice were kept in an air-filtered, light-controlled room, at constant temperature (22–24°C), with humidity between 40 and 70%, and were permitted free access to a standard diet.

### Establishment of ALI model induced by LPS in mice

LILRB4 KO mice were obtained from RIKEN BioResource Center (Stock Number 02692) [12,15]. The (LILRB4 KO and WT mice (*n*=6–10) were used to construct the ALI animal model. The experimental mice were anesthetized with tribromoethanol. When their respiration was uniform and slow, they were injected with 50 µl of LPS (30 μg/g) via a high-pressure air mist microsyringe, after which the mice were kept in position for 5 min to prevent liquid reflux. Mice were then laid on their backs in 28°C warm box until they recovered from anesthesia. After 6 h of LPS stimulation, we performed intraperitoneal injections of pentobarbital sodium (50 mg/kg) to anesthetize the mice. Venous serum, total lung irrigation solutions, left lung tissue and right lung tissue were collected successively for various tests.

### Bone marrow cell transplantation

The recipient mice (WT and LILRB4 KO mice) were irradiated twice (600 cGy at an interval of 5 h) to kill the bone marrow hematopoietic stem cells. Marrow cell suspensions were obtained from donor mice and each recipient mouse was given a dose of 1 × 10^7^ bone marrow cells via venous infusion. After 6–8 weeks, immune cells differentiated from the donor bone marrow cells almost completely replaced the peripheral white blood cells and lung immune cells of the recipient mice. At this point, ALI was induced by LPS and the corresponding indicators were detected.

### Isolation and culture of primary alveolar macrophages

Mice were killed by cervical dislocation and disinfected with 75% ethanol. The mice were then fixed on sterile paper in a supine position. Sterile surgical scissors were used to cut away the neck skin of the mice along the median line to separate out the trachea. The cephalic trachea was ligated under the cricoid cartilage and a ‘V’ incision was cut into the middle of the trachea. We injected 1 ml of phosphate buffered saline (PBS) into the lung cavity, gently massaged the lung for 1 min, and then slowly pulled off the bronchial alveolar lavage solution. The above procedure was repeated 15 times and approximately 10–15 ml of liquid was collected. The liquid was centrifuged at 300×***g*** for 5 min and the upper suspension was discarded. After complete lysis of red blood cells using a red blood cell lysis buffer (Bioer, BSA06M1, China), RPMI-1640 culture medium was used to resuspend and count the cells. Trypan Blue staining was used to test cell viability and the cell density was adjusted to 5 × 10^5^ cells/ml. The cells were inoculated into six-well plates and cultured overnight, after which the nonadherent cells were removed and discarded.

### Isolation and culture of primary bone marrow-derived macrophages

Mouse macrophages were collected from bone marrow suspensions harvested from mice aged for 6–8 weeks according to previously described methods, with some modifications. Briefly, bone marrow cells were harvested from the femurs and tibias of mice, followed by red blood cell depletion and 2× PBS washes. Cells were resuspended in RPMI-1640 containing 10% heat inactivated FCS and 30 ng/ml M-CSF, followed by inoculation into six-well plates at a density of 1 × 10^5^ cells/well in 2 ml/well. After 2 and 4 days of culture, the floating granulocytes were gently removed and fresh M-CSF-containing medium was added.

To establish the *in vitro* ALI model, primary alveolar macrophages (AMs) and primary bone marrow macrophages were treated with LPS (100 ng/ml) for the indicated amounts of time.

### Hematoxylin and Eosin staining

The lungs were excised and fixed in 10% phosphate-buffered formalin, embedded in paraffin, and sectioned into 4-mm thick sections according to standard procedures. We deparaffinized and gradually hydrated the sections before examining them by Hematoxylin and Eosin (H&E) staining. A pathologist who was blind to the experimental protocol provided the morphological assessments.

### Immunohistochemistry

We prepared sections according to the manufacturer’s guidelines for immunohistochemical assays. We analyzed and evaluated CD11b (ab75476; Abcam, Cambridge Science Park, U.K.) and Ly6G (551459; BD Biosciences) expression based on the staining intensity by microscopy.

### Enzyme-linked immunosorbent assay

Bronchoalveolar lavage fluid (BALF) was harvested and used for enzyme-linked immunosorbent assays (ELISAs). Mouse TNF-α (900-T54; Peprotech), IL-6 (m6000b; R&D), and total protein and IgM (E99-101; Bethyl) Quantikine ELISA kits were used to detect TNF-α, IL-6, total protein and IgM, according to the manufacturer’s instructions.

### Western blot

Proteins were extracted from lung tissues, AMs and bone marrow-derived macrophages (BMDMs) according to standard protocols that the tissue or cells are ground and then RIPA lysate (50 mM Tris/HCl pH 7.4, 150 mM NaCl, 1% Triton X-100 or NP-40, 1% sodium deoxycholate, 0.1% SDS, 1 mM EDTA) containing PMSF is added to acquire protein. Protein concentrations were determined using a BCA Protein Assay kit. In brief, the protein samples were separated on a 12.5% sodium dodecyl sulfate/polyacrylamide gel and then transferred to a nitrocellulose membrane. The membrane was blocked using 5% nonfat dry milk in a TBS-T buffer and then incubated overnight at 4°C with primary antibody. After rinsing the blots extensively with TBS-T buffer, they were incubated with HRP–conjugated secondary antibodies, developed using an enhanced chemiluminescence system, and captured on light-sensitive imaging film.

### PCR

TRIzol reagent (Invitrogen) was used to extract total RNA from cultured AMs, primary BMDMs and lung tissues according to the manufacturer’s instructions. After the RNA was reverse transcribed into cDNA using the Transcriptor First Strand cDNA Synthesis Kit (04896866001; Roche, Basel, Switzerland), quantitative real-time PCR amplification was performed using a SYBR Green PCR Master Mix. The PCR conditions were as follows: 95°C for 10 min; 40 cycles of 95°C for 10 s, 60°C for 10 s, and 72°C for 20 s; a final extension at 72°C for 10 min. Each experiment was performed in triplicate and the results were determined as the average gene expression normalized to β-actin expression. The primers used are described in Table 1. The relative mRNA expression levels were calculated using the 2^−ΔΔ*C*^_t_ method and were normalized against β-actin.

**Table 1:** Primer information

Gene	Forward primer	Reverse primer
*LILRB4*	TGCTTTTTCTCATCCTCATCGGA	GTGGGTTCCAACTGTTCAGC
*IL1β*	CCGTGGACCTTCCAGGATGA	GGGAACGTCACACACCAGCA
*IL6*	AGTTGCCTTCTTGGGACTGA	TCCACGATTTCCCAGAGAAC
*Ccl2*	TACAAGAGGATCACCAGCAGC	ACCTTAGGGCAGATGCAGTT
*TNF-α*	CATCTTCTCAAAATTCGAGTGACAA	TGGGAGTAGACAAGGTACAACCC
*β-actin*	GTGACGTTGACATCCGTAAAGA	GCCGGACTCATCGTACTCC

### Statistical analysis

All data are presented as the mean ± standard error. We used Statistical Product and Service Solutions (SPSS) 19.0 for all statistical analysis. Statistical differences among more than two groups were compared using a one-way ANOVA, followed by Bonferroni analyses (for data meeting the homogeneity of variance) or Tamhane’s T2 analyses (for data demonstrating heteroscedasticity). Statistical differences between two groups were compared with a two-tailed Student’s *t*test. *P*<0.05 was considered significant.

## Results

### The expression of LILRB4 is increased in the lung after ALI

**Figure 1:**
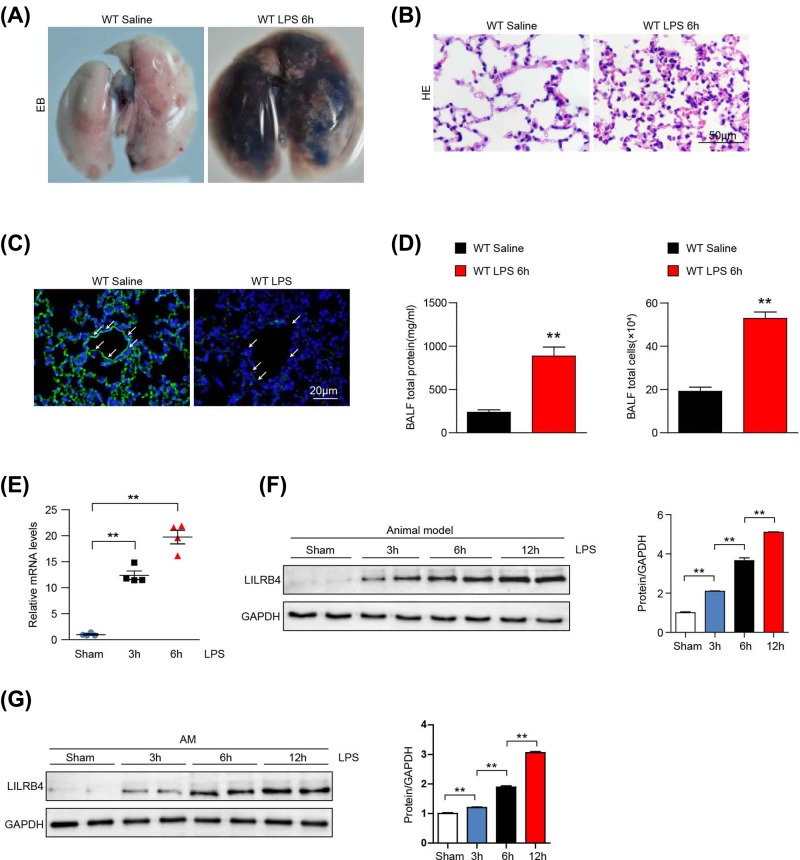
LILRB4 expression in lung tissue from a mouse model of LPS-induced ALI (**A**) Evans blue staining images of lung tissues after LPS or saline stimulation. (**B**) Lung tissue pathology in WT mice treated with LPS or saline stimulation. (**C**) The structure of endothelial cells in WT mice treated with LPS or saline stimulation. (**D**) Total protein and total cell numbers in the BALF from the mice treated with LPS or saline stimulation. (**E**) LILRB4 mRNA expression in lung tissues after LPS stimulation (*n*=4 per group). (**F**) LILRB4 protein expression in lung tissues after LPS stimulation for 3, 6 and 12 h (*n*=2 per group). (**G**) Western blot of LILRB4 protein expression in primary AMs after LPS stimulation for 3, 6 and 12 h. The results shown are representative of three blots. For (F,G), GAPDH served as loading controls. A one-way ANOVA was used for statistical analysis of (A–C). ^**^P<0.01.

To clarify the potential function of LILRB4 in ALI, ALI animal models with LPS stimulation at different times were constructed [16]. The ALI model features were verified at different levels. Evans Blue staining showed that the lung of mice after treatment with LPS is bluer than that of sham group (Figure 1A). The HE stain also showed more inflammatory cell infiltration and tissue damage in the lung tissue from the mice treated with LPS (Figure 1B).CD31 was used as a marker of vascular endothelial cells and the immunofluorescence staining showed that the structure of endothelial cells was destroyed significantly compared with sham group (Figure 1C). In addition, protein content and total cells in the BALF of mice after treating with LPS is significantly increased compared with sham group (Figure 1D), after the model of ALI was successfully constructed. As shown in Figure 1E, LILRB4 mRNA expression levels were increased significantly in lung tissues at 3 h after LPS stimulation, with even higher expression at 6 h. Consistently, LILRB4 protein expression was also gradually up-regulated over time in lung tissue after LPS stimulation (Figure 1F). More importantly, we isolated primary AMs and treated them with LPS and found that the expression of LILRB4 was increased significantly in AMs after LPS stimulation for 3, 6 and 12 h, and this expression was dependent on time (Figure 1G). These results suggest that LILRB4 may play an important role in ALI.

### LILRB4 deficiency exacerbates LPS-induced ALI

**Figure 2:**
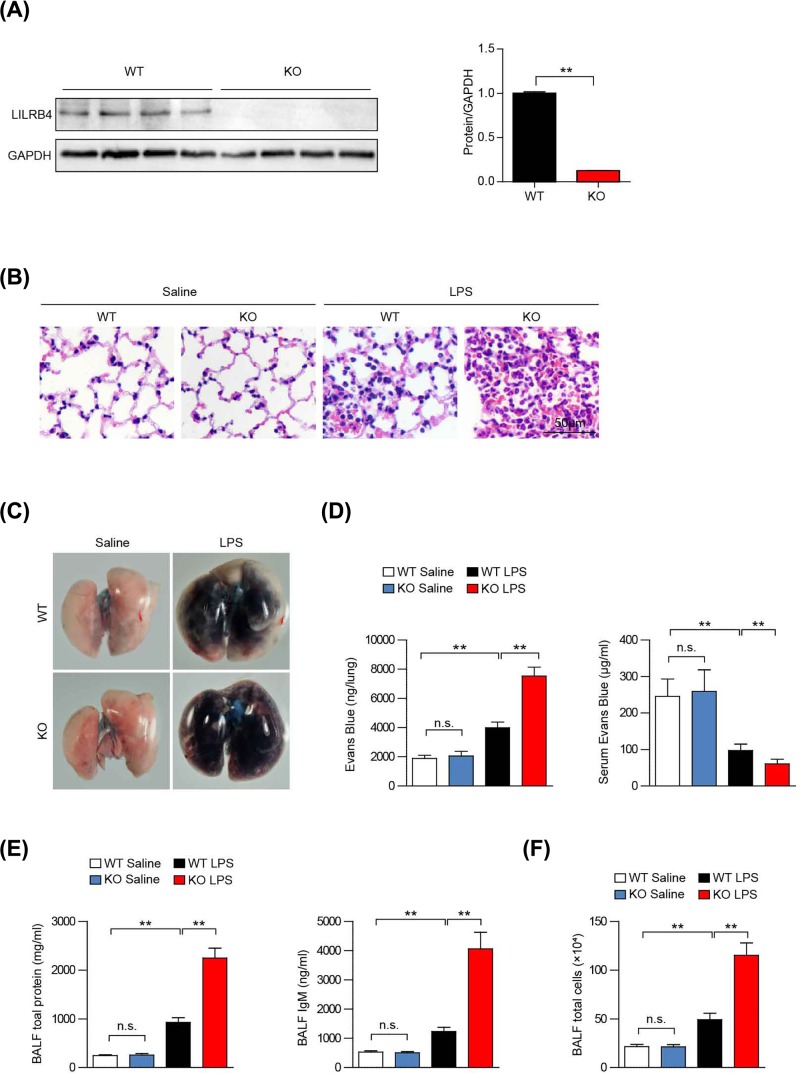
LILRB4 deficiency exacerbates LPS-induced ALI (**A**) Western blot of LILRB4 expression in knockout and WT mice (*n*=3 per group). GAPDH served as a loading control. (**B**) Lung tissue pathology in knockout and WT mice after LPS stimulation (*n*=6 per group). (**C**) Knockout and WT mouse lung images after LPS stimulation (*n*=6 per group). (**D**) Evans Blue contents in lung tissue and serum of WT and LILRB4 KO mice after LPS stimulation (*n*=6 per group). (**E**) Total protein and IgM content in the BALF from the KO and WT mice stimulated with LPS (*n*=8 per group). (**F**) Total cell numbers in the BALF from the KO and WT mice stimulated with LPS (*n*=8 per group). For statistical analyses, a two-tailed Student’s *t* test was used for (A) and one-way ANOVAs were used for (D–F). Abbreviation: n.s., not significant; ^**^P<0.01.

To explore the function of LILRB4 in ALI, we administered LPS or saline into the lungs of LILRB4 KO and wild-type littermates (WT) through intratracheal injection using established protocols (Figure 2A). Compared with saline controls, the lung tissues of WT and LILRB4 KO mice showed pathological manifestations of alveolar interstitial thickening, inflammatory cell and erythrocyte infiltration, and alveolar structural destruction after LPS stimulation. However, LILRB4 KO mice showed more serious lung tissue structural damage than WT mice (Figure 2B). Next, we measured alveolar air–blood barrier permeability by detecting Evans Blue penetration. The results shown in Figure 2C indicated that LILRB4 KO mice exhibited enlarged lung tissues with a darker color indicating that the lung tissues were filled with more Evans Blue compared with WT mice. Consistent with this, the amount of Evans Blue in the lung tissue was significantly increased, while the amount in the serum was significantly decreased in LILRB4 KO mice compared with WT mice (Figure 2D). After ALI, alveolar–capillary integrity is impaired, allowing proteins in the blood to seep into the alveoli, leading to the accumulation of protein-rich fluid in the alveoli [17]. Therefore, we measured the protein content in the BALF. Figure 2E,F show that the total protein and IgM content and total cell numbers in the BALF were also significantly increased in the KO mice compared with the WT mice. These results indicate that LILRB4 deficiency exacerbates LPS-induced ALI.

### LILRB4 deficiency promotes inflammatory cell infiltration and cytokine production in LPS-induced ALI

**Figure 3:**
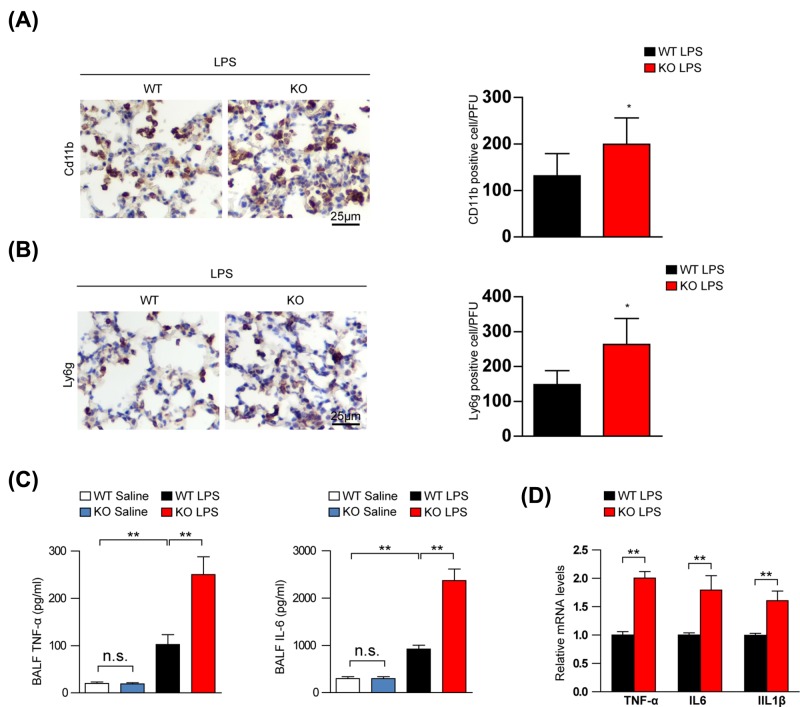
LILRB4 deficiency induces inflammation in LPS-induced ALI (**A**) Immunohistochemistry analysis of CD11b positive cells in lung tissue from WT and KO mice stimulated with LPS (*n*=6 per group). (**B**) Immunohistochemistry analysis of Ly6G positive cells in lung tissue from WT and KO mice stimulated with LPS (*n*=6 per group). (**C**) Amount of TNF-α and IL-6 in the BALF of WT and KO mice stimulated with LPS or saline for 6 h (*n*=8 per group). (**D**) Cytokine mRNA levels were determined by quantitative real-time PCR in lung tissues of KO and WT mice stimulated with LPS for 6 h (*n*=4 per group). For statistical analyses, a one-way ANOVA was used for (C) and the two-tailed Student’s *t*test was used for (A,B,D). Abbreviation: n.s., not significant; ^*^P<0.05, ^**^P<0.01.

One of the main mechanisms of ALI pathogenesis is inflammation [18]. Thus, we investigated the effects of LILRB4 on the inflammation induced by LPS. We found that LILRB4 deficiency significantly increased inflammatory cell infiltration (CD11b^+^ cells and Ly6G^+^ cells) in the lung tissues (Figure 3A,B). TNF-α, IL-1β, and IL-6 are characteristic cytokines involved in the inflammatory process of ALI [18–20]. The amount of TNF-α and IL-6 in the BALF were significantly increased in the LILRB4 KO mice compared with the WT mice (Figure 3C). Additionally, the expression of TNF-α, IL6 and IL1β mRNAs were up-regulated in the lungs of LILRB4 KO mice (Figure 3D). Taken together, these results demonstrate that LILRB4 deficiency promotes inflammatory cell infiltration and cytokine production in ALI.

### ALI-associated inflammation is dependent on BMDMs

**Figure 4:**
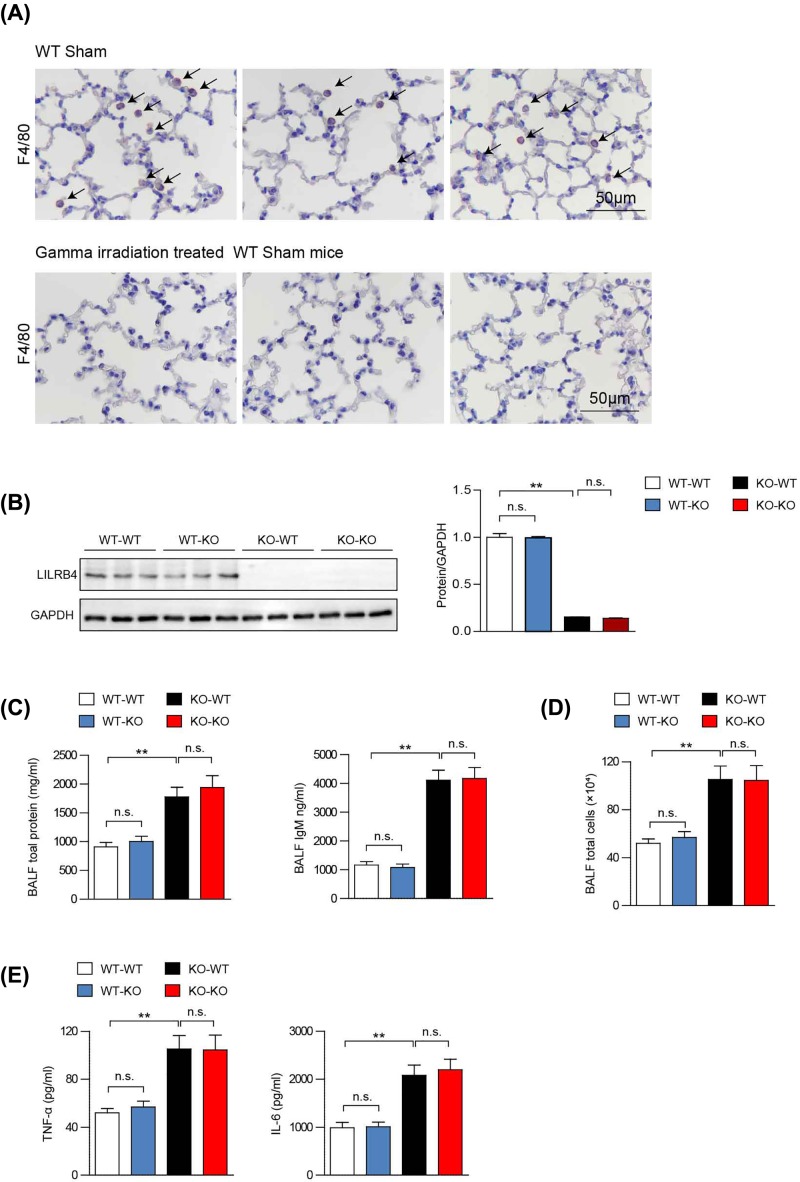
LILRB4-induced inflammation in ALI is dependent on bone marrow-derived cells (**A**) The immunohistochemical analysis of F4/80 macrophage in the lung tissue from WT sham group and γ irradiation treated WT sham mice. (**B**) Western blot analysis of LILRB4 in primary BMDMs after bone marrow cell transplants between KO and WT mice (*n*=3 per group). GAPDH served as the loading control. (**C**) Total protein and IgM content in BALF of bone marrow cell transplant mice after stimulation with LPS (*n*=10 per group). (**D**) Total cell numbers in the BALF of the experimental group (*n*=10 per group). (**E**) Contents of TNF-α and IL-6 in the BALF (*n*=10 per group). For statistical analyses, one-way ANOVAs were used for (A–D). Abbreviation: n.s., not significant; ^**^P<0.01.

To clarify whether LILRB4 regulates ALI through bone marrow-derived immune cells or lung parenchyma cells, we performed bone marrow transplants in WT and LILRB4 KO mice after γ irradiation. To verify whether γ irradiation for BM ablation was effective, we performed immunohistochemical analysis of F4/80 to detect macrophage. The results showed that the lung had absence of AM after γ irradiation, indicating that γ irradiation for BM ablation in our study was effective (Figure 4A). WT bone marrow was transplanted into LILRB4 KO recipients (WT-KO) and control WT recipients (WT-WT), and LILRB4 KO bone marrow was transplanted into WT recipients (KO-WT), as well as into LILRB4 KO recipients as a control (KO-KO). In the present study, we used the same batches of mice of the same weight and age, and we infused with the same amount of bone marrow cells each time according to the standard procedure. Two sentinel mice were set up in each batch of irradiated mice. The mice were not injected with donor bone marrow after irradiation, and all died within a week of irradiation, suggesting that the recipient bone marrow was completely cleared by irradiation. In contrast, all the mice receiving donor bone marrow survived, suggesting that the donor bone marrow was successfully implanted. In addition, we isolated protein from the blood of each batch of mice, and Western blot results showed that LILRB4 was present in WT-KO group, whereas was absent from KO-WT group, indicating that BM transplants were effective and consistent. Successful bone marrow implantation and chimerism were examined by Western blot (Figure 4B). Compared with the WT-WT group, the total protein and IgM contents in the BALF of the KO-WT group were significantly increased (Figure 4C). Under normal conditions, the number of cells in the BALF is very low, but under pathological conditions such as inflammation, a large number of inflammatory cells migrate to the lungs to form an exudate. Therefore, the total number of cells in the BALF can reflect the severity of the inflammatory injury. We quantitated the total number of cells in the BALF and found that the total number of cells in the BALF of the KO-WT group were significantly increased (Figure 4D). The classical inflammatory cytokines, TNF-α and IL-6, were significantly up-regulated in the BALF of the KO-WT group (Figure 4E). However, there were no significant differences between the WT-WT and the WT-KO groups, or between the KO-WT and the KO-KO groups (Figure 4C–E). These results suggest that bone marrow-derived leukocytes, but not lung stromal cells, are required for the LILRB4-mediated pulmonary inflammatory response in response to LPS stimulation.

### LILRB4 regulates the BMDMs inflammatory response induced by LPS stimulation

Next, we tested the role of LILRB4 in regulating the macrophage inflammatory response induced by LPS stimulation.

**Figure 5:**
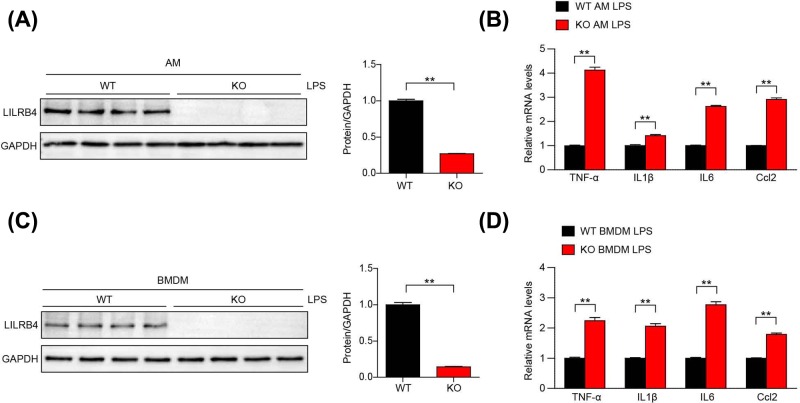
LILRB4-induced inflammation in ALI is dependent on BMDMs (**A**) Western blot analysis of LILRB4 expression in AMs isolated from WT and LILRB4 KO mice. (**B**) TNF-α, IL1β, IL6 and Ccl2 mRNA levels were determined by quantitative real-time PCR in LPS-stimulated AMs from KO and WT mice. (**C**) Western blot analysis of LILRB4 expression in BMDMs isolated from WT and LILRB4 KO mice. (**D**) TNF-α, IL1β, IL6 and Ccl2 mRNA levels were determined by quantitative real-time PCR in LPS-stimulated BMDMs from KO and WT mice. For (A,C), results shown are representative of three blots, and GAPDH served as the loading control. For (B,D), the results shown are representative of three independent experiments. For statistical analyses, a two-tailed Student’s *t*test was used for (A–D). ^**^P<0.01.

We isolated the primary AMs and BMDMs from WT and LILRB4 KO mice and verified that LILRB4 was silenced by Western blot (Figure 5A,C). Then we quantitated classical cytokines and chemokines. Compared with the WT AMs treated with LPS, the LILRB4 KO AMs treated with LPS had increased expression of TNF-α, IL1β, IL6 and Ccl2 (Figure 5B). Consistent with this result, we treated primary BMDMs from WT and LILRB4 KO mice with LPS and found that these inflammatory cytokines were also up-regulated in LILRB4 KO BMDMs (Figure 5D). These results suggest that one of the main mechanisms by which LILRB4 regulates LPS-induced ALI is by regulating the inflammatory response of macrophages.

### BMDM-dependent inflammation in ALI occurs via the NF-κB pathway

**Figure 6:**
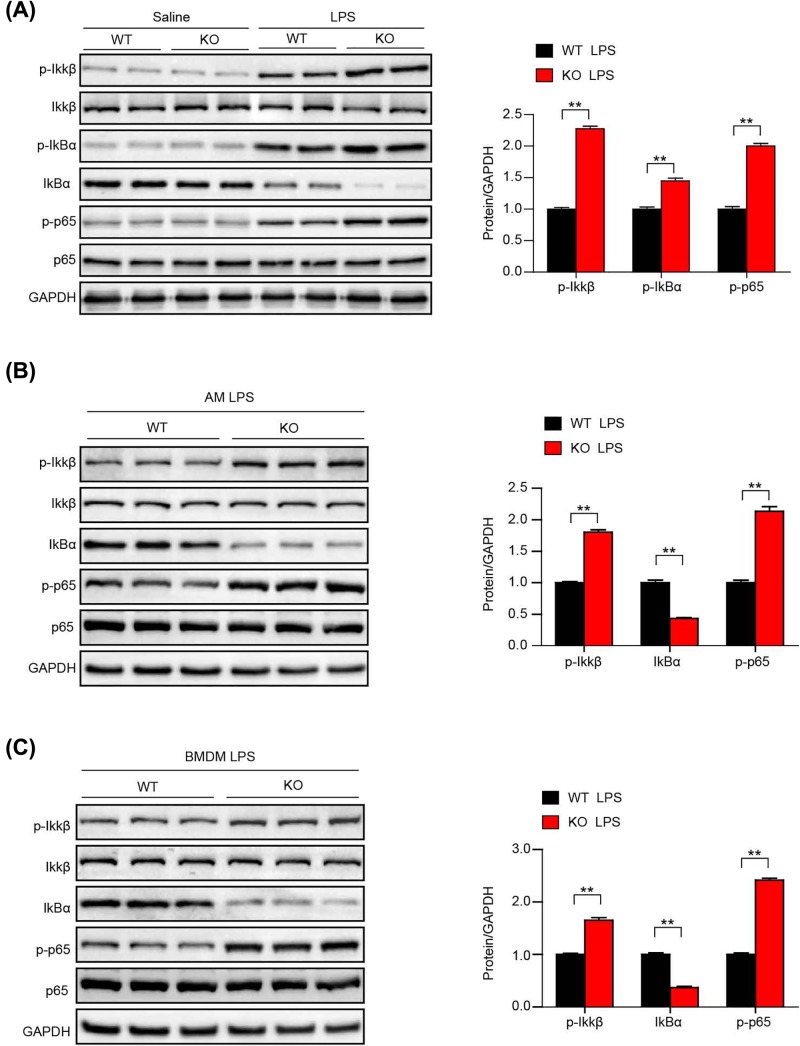
LILRB4 deficiency increases the inflammatory response in ALI via the NF-κB signaling pathway in macrophages (**A**) Western blot analysis of NF-κB signaling in LILRB4 KO or WT mice treated with LPS for 6 h. (**B**) Western blot analysis of NF-κB signaling in AMs from LILRB4 KO or WT mice stimulated with LPS for 6 h. (**C**) Western blot analysis of NF-κB signaling in BMDMs from LILRB4 KO or WT mice stimulated by LPS for 6 h. For (A–C), the results shown are representative of three blots, and GAPDH served as the loading control. For statistical analyses, a two-tailed Student’s *t*test was used for (A–C). ^**^P<0.01.

To further validate the molecular mechanism of the role of LILRB4 in regulating the inflammatory response of macrophages, we assessed the activation of the classical inflammatory pathway, the NF-κB pathway prior to bone marrow transplant [21]. Western blot analysis showed that the classical NF-κB proinflammatory signaling pathway was dramatically activated in LILRB4-deficient mice, as demonstrated by significant increases in the activation of the p-IKKβ, p-p65 and p-IκBα proteins (Figure 6A). To explore the NF-κB signaling pathway in macrophages, AMs and BMDMs were stimulated with LPS. Similar to our *in vivo* results, Western blot analysis showed that the NF-κB proinflammatory signaling pathway was dramatically activated in LILRB4-deficient macrophages stimulated with LPS, as demonstrated by significant increases in the activation of the p-IKKβ, p-p65 and decreased IkBα protein levls(Figure 6B,C). These data indicate that the macrophage-dependent inflammatory responses prompted by LILRB4 deficiency during LPS-induced ALI occur via the NF-κB pathway.

## Discussion

At present, the clinical treatment for ALI/ acute respiratory distress syndrome (ARDS) mainly consists of symptomatic support therapies, such as improving ventilation, correcting acid–base balance disorders and maintaining humoral balance [1]. However, the lack of treatments or drugs that are specific to the mechanisms of ALI pathogenesis has led to a high morbidity and mortality. There are 190000 new cases of ALI in America every year and approximately 75000 deaths [15]. The pathogenesis of ALI/ARDS has not been fully characterized. Damage of alveolar–capillary barrier, inflammation, oxidative injury and lung cell apoptosis represent the key features of ALI [22].

LILRB4 was first identified as an inhibitory receptor expressed on myeloid antigen-presenting cells that associate with the protein tyrosine phosphatase SHP-1 [10]. Previous studies have shown that LILRB4 plays an important role in the regulation of natural immunity and inflammation [14]. LILRB4 inhibits IgE-dependent activation of mast cells, NK cell activation and contributes inhibition of neutrophil-dependent inflammation *in vitro* [14]. LILRB4 can recruit the SH2-containing tyrosine kinase-1 protein through its ITIM structure, inhibit the transcriptional activation of NF-κB, and thus inhibit an overactivated inflammatory response [23,24]. The inflammation in ALI is involved with multiple immune cells including T cells, mast cells, neutrophils, DCs and macrophage [25–27], but macrophage is the main immune cell located in the lung tissue and other immune cells are derived from the extrapulmonary environment. In our study, we constructed the ALI model with mice treated with LPS. LILRB4 mRNA and protein expression levels were significantly increased and mainly concentrated in macrophages in the lung tissues of mice with ALI, suggesting that LILRB4 may be involved in the occurrence and development of LPS-induced ALI (Figure 1).

LILRB4 in uterine macrophages can inhibit the production of TNF-β, which is mediated by the cross-linking of the Fc-α receptor-I, and plays an important role in maternal immune tolerance [28]. In addition, LILRB4 can inhibit the activation of auxiliary T cells, inactivate activated effector cells and transform them into inhibitive T cells and regulatory T cells, and play an important role in transplant tolerance, hypersensitivity and autoimmune diseases, such as lupus erythematosus [13,29–31]. To test the LILRB4 in ALI, we designed LILRB4 KO mice and found that LILRB4 deficiency exacerbated lung injury in ALI model (Figure 2).

The mechanism of ALI is associated with many factors, such as inflammation, immune responses, and reactive oxygen species, among others [32]. However, inflammation is a core aspect of ALI. There has been much evidence suggesting that LILRB4 ablation can cause immune imbalances and exacerbate inflammatory disease. Soluble LILRB4 has been found in the serum of patients with melanoma, colorectal cancer and pancreatic cancer. This soluble LILRB4 can damage T-cell responses and block LILRB4 signaling, which is crucial to immunotherapy success in the treatment of malignant tumors [13,23,29]. LILRB4 expression is up-regulated in atherosclerotic lesions in human coronary arteries and in mouse aortic roots, and LILRB4 deletion significantly accelerated the development of atherosclerotic lesions [12]. LILRB4 inhibits the expression of TRAF6 and downstream inflammatory factors, thereby inhibiting the occurrence and development of non-alcoholic fatty liver disease [33]. Our results also provide evidence that LILRB4 KO prompted typical inflammatory phenomena, including increased neutrophil and macrophage infiltration and an up-regulation of cytokine and chemokine secretion (Figure 3).

Macrophages are a type of immune cell with vital importance in ALI development [34,35]. LPS stimulates the immune response and induces the secretion of chemokines that recruit monocytes to the pulmonary alveoli where they differentiate into macrophages. Previous studies have elucidated the effects of LILRB4 on LPS-induced ALI; however, conventional LILRB4 KO mice were applied in the present study, and it was not clear whether LILRB4 regulated the occurrence and development of ALI by regulating the function of pulmonary immune cells or pulmonary parenchymal cells (epithelial/endothelial cells). To clarify this issue, we used bone marrow transplantation to explore the cell source of LILRB4-regulated ALI. The bone marrow transplant results demonstrated that LILRB4 regulates the development of LPS-induced ALI mainly through immune cells derived from the bone marrow (Figure 4). The immune cells associated with ALI include macrophage, T cell, dendritic cell and other immune cell [16,20,26,35]. LILRB4 is a potent inhibitory regulator of LPS-induced, neutrophil-dependent inflammation. Previous study discovered a regulatory role for LILRB4 on DCs in a mouse model of Th2 pulmonary inflammation induced by inhalation sensitization with OVA and LPS [36,37]. In the present study, we showed that LILRB4 regulated ALI mainly through its function in macrophages. However, we could not exclude the potential effect of other immune cell in the regulation of LILRB4 in the pathogenesis of ALI (Figure 5).

NF-κB is a protein complex composed of a family of inducible transcription factors that play a variety of evolutionarily conserved roles in the immune system [21,38]. NF-κB is required for maximal transcription of numerous cytokines, including TNF-α, IL-1β and IL-6, all of which are very important in regulating inflammatory responses during the development of ALI [20,21]. As a result, inhibition of the NF-κB pathway is a key step in relieving the inflammatory response during ALI and other pulmonary diseases [39,40]. *In vivo* and *in vitro* analyses revealed that the proinflammatory effects of LILRB4 deficiency were mediated by the increased activation of NF-κB signaling. Together these results indicated that the LILRB4-induced exacerbated inflammatory response in ALI was dependent on BMDMs via the NF-κB pathway (Figure 6). As we know, there is a cross-talk between antigen-presenting cells and lymphocytes involved in immune suppression. The interaction between LILRB4 and its still unidentified ligand on the surface of activated human T cells is potentially important for immune regulation network [41]. Since the ligand of LILRB4 is still unclear, the molecular mechanism of LILRB4 in macrophages still needs to be further studied.

In conclusion, our study revealed that LILRB4 in macrophages can regulate the development of inflammation via regulating the NF-κB-mediated inflammatory response. Therefore, targeting the LILRB4 pathway may be a novel therapeutic target for ALI.

## Abbreviations

ALI: acute lung injury
AM: alveolar macrophage
ARDS: acute respiratory distress syndrome
BALF: bronchoalveolar lavage fluid
BMDM: bone marrow-derived macrophage
DC: dendritic cell
ELISA: enzyme-linked immunosorbent assay
FCS: fetal calf serum
ICAM-1: Intercellular adhesion molecules 1
ITIM: immunoreceptor tyrosine-based inhibitory motif
LILRB4: leukocyte immunoglobulin-like receptor B4
LILRB4 KO: LILRB4 knockout
LPS: lipopolysaccharide
MCP-1: Monocyte chemoattractant protein-1
NK: natural killer
PBS: phosphate buffered saline
